# In vitro–transcribed guide RNAs trigger an innate immune response via the RIG-I pathway

**DOI:** 10.1371/journal.pbio.2005840

**Published:** 2018-07-16

**Authors:** Beeke Wienert, Jiyung Shin, Elena Zelin, Kathleen Pestal, Jacob E. Corn

**Affiliations:** 1 Innovative Genomics Initiative, University of California, Berkeley, Berkeley, California, United States of America; 2 Department of Molecular and Cell Biology, University of California, Berkeley, Berkeley, California, United States of America; National Cancer Institute, United States of America

## Abstract

Clustered, regularly interspaced, short palindromic repeat (CRISPR)–CRISPR-associated 9 (Cas9) genome editing is revolutionizing fundamental research and has great potential for the treatment of many diseases. While editing of immortalized cell lines has become relatively easy, editing of therapeutically relevant primary cells and tissues can remain challenging. One recent advancement is the delivery of a Cas9 protein and an in vitro–transcribed (IVT) guide RNA (gRNA) as a precomplexed ribonucleoprotein (RNP). This approach allows editing of primary cells such as T cells and hematopoietic stem cells, but the consequences beyond genome editing of introducing foreign Cas9 RNPs into mammalian cells are not fully understood. Here, we show that the IVT gRNAs commonly used by many laboratories for RNP editing trigger a potent innate immune response that is similar to canonical immune-stimulating ligands. IVT gRNAs are recognized in the cytosol through the retinoic acid–inducible gene I (RIG-I) pathway but not the melanoma differentiation–associated gene 5 (MDA5) pathway, thereby triggering a type I interferon response. Removal of the 5’-triphosphate from gRNAs ameliorates inflammatory signaling and prevents the loss of viability associated with genome editing in hematopoietic stem cells. The potential for Cas9 RNP editing to induce a potent antiviral response indicates that care must be taken when designing therapeutic strategies to edit primary cells.

## Author summary

Clustered, regularly interspaced, short palindromic repeat (CRISPR)–CRISPR-associated 9 (Cas9) genome editing is transforming fundamental research, as it allows researchers to make targeted changes to the genome of cells. For efficient editing, the Cas9 protein (a DNA nuclease) and a guide RNA (gRNA), which leads the nuclease to the correct location in the genome, have to be introduced into cells. One recent advancement is the delivery of a Cas9 protein and an in vitro–transcribed (IVT) gRNA as a precomplexed ribonucleoprotein (RNP). This approach allows editing of more sensitive cell types such as immune cells and hematopoietic stem cells. However, the consequences of introducing foreign Cas9 nuclease and gRNA into mammalian cells are not fully understood. Here, we show that in many cell types, the IVT gRNAs trigger a potent innate immune response—a natural defense mechanism against RNA viruses. We show that the innate immune response causes cell death in primary hematopoietic stem cells but that removal of the 5’-triphosphate from gRNAs by phosphatase treatment can ameliorate the immune response and prevent the loss of viability. Hence, CRISPR-Cas9 RNP editing has the potential to induce a potent antiviral response, and we suggest that care must be taken when designing therapeutic strategies to edit primary cells.

## Introduction

Clustered, regularly interspaced, short palindromic repeat (CRISPR)–CRISPR-associated (Cas) genome editing has rapidly become a widely used tool in molecular biology laboratories. Its ease of use and high flexibility allows researchers to modify and edit genomes in cell lines [1], stem cells [2], animals and plants [3,4], and even human embryos [5]. The Cas protein complexes with a target-specific CRISPR RNA (crRNA) and a *trans*-activating crRNA (tracrRNA), which keeps the Cas protein catalytically active [6]. In experimental procedures, the two RNAs are often combined to generate a single guide RNA (gRNA), which means that at least two components must be successfully delivered into cells during genome editing: the Cas protein, such as Cas9, and gRNA to direct the Cas9 protein to its target site. For in vitro–cultured cells, this can be done by transfecting plasmids encoding gRNA and Cas9 protein. However, transfection of plasmid DNA into sensitive cell types such as primary and stem cells is challenging and inefficient. The introduction of plasmids can also lead to undesired integration of DNA at the cut site [7], increased off-target activity through prolonged expression of the CRISPR-Cas9 components [8], and a delay in editing while the cell expresses gRNA and Cas protein [9].

The delivery of gRNA and Cas9 protein as a precomplexed ribonucleoprotein (RNP) sidesteps issues related to plasmid expression and has proved to be a successful strategy to edit human primary cells, including T cells [10,11], hematopoietic stem cells [12–15], and neurons [16]. This makes RNP editing a particularly attractive approach for therapeutic applications, but relatively little is known about the nonediting consequences of introducing a foreign gRNA and Cas9 protein. Human cells have evolved multiple defense mechanisms to guard against foreign components, and genome editing reagents have the potential to activate these systems. For example, recent data suggest that humans may have a preexisting adaptive immune response to the Cas9 protein [17]. But cellular responses to the gRNAs used to program Cas9 editing have so far not been well explored.

Cells respond to infection by RNA viruses with an innate immune response that protects the host cell from invading foreign genetic material [18]. Foreign RNAs are recognized by pathogen-associated molecular pattern (PAMP) binding receptors in the cytosol that include retinoic acid–inducible gene I (RIG-I) and melanoma differentiation–associated gene 5 (MDA5) [19]. This triggers a cascade of events mediated by the mitochondrial antiviral signaling (MAVS) protein, resulting in the transcriptional activation of type I interferons and interferon-stimulated genes (ISGs) [20–22]. RNA PAMPs usually contain exposed 5’-triphosphate ends [19], which may also be present in gRNAs made via T7 in vitro transcription [23,24]. Given that Cas9 has a picomolar affinity for targeting gRNA [25], it is not clear that the 5’-triphosphate would be available to stimulate an innate immune response.

We asked whether in vitro-transcribed (IVT) gRNAs complexed with Cas9 cause an innate immune response and here show that introduction of RNPs into cells induces up-regulation of interferon beta (IFNβ) and interferon-stimulated gene 15 (ISG15) in a variety of human cell types. This activity depends upon RIG-I and MAVS but is independent of MDA5. The extent of the immune response depends upon the protospacer sequence, but removal of the 5’-triphosphate from gRNAs avoids stimulation of innate immune signaling. The potential for Cas9 RNP editing to induce an antiviral response indicates that care must be taken when designing therapeutic strategies to edit primary cells.

## Results

**Fig 1:**
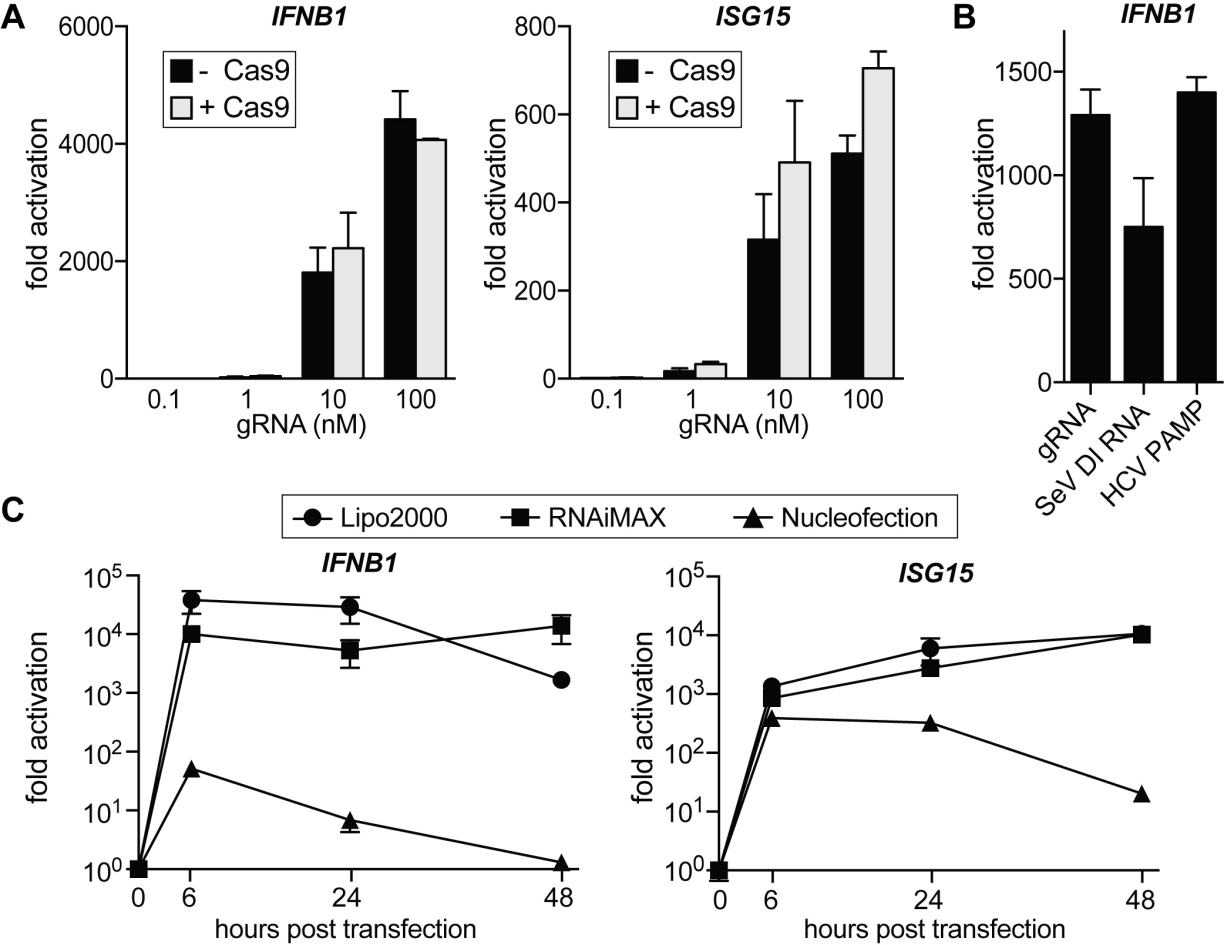
Transfection of IVT gRNAs into HEK293 cells triggers a type I interferon response. (A) qRT-PCR analysis of *IFNB1* and *ISG15* transcript levels in HEK293 cells transfected with increasing amounts of gRNA with and without Cas9 protein. In the samples with Cas9, gRNAs were complexed with constant amounts (100 pmol, 100 nM final concentration) of Cas9 protein. Cells were harvested for RNA extraction 30 h after transfection using CRISPRMAX transfection reagent. Ct values were normalized to Ct values of mock-transfected HEK293 cells to determine fold activation. (B) qRT-PCR analysis of *IFNB1* transcript levels in HEK293 cells transfected with equimolar amounts (50 nM) of IVT gRNA, SeV DI RNA, or HCV PAMP, respectively. (C) qRT-PCR analysis of *IFNB1* and *ISG15* transcript levels in HEK293 cells over a 48-h time course after transfection with 50 nM via lipofection (Lipofectamine2000 or RNAiMAX) or nucleofection, respectively. For all panels, average values of 3 biological replicates +/−SD are shown. The underlying data for this figure can be found in S1 Data. Cas9, CRISPR-associated 9; Ct, cycle threshold; gRNA, guide RNA; HCV, hepatitis C virus; HEK 293, human embryonic kidney 293; *IFNB1*, *interferon beta 1*; IVT, in vitro–transcribed; PAMP, pathogen-associated molecular pattern; qRT-PCR, quantitative real-time PCR; SeV DI, Sendai virus defective interfering.

To investigate if mammalian cells react to IVT gRNA/Cas9 with an innate immune response, we first performed genome editing in human embryonic kidney 293 (HEK293) cells using Cas9 RNPs. To separate innate immune response from genome editing, we performed these experiments with a nontargeting gRNA that recognizes a sequence within blue fluorescent protein (BFP) and has no known targets within the human genome [26]. Constant amounts of recombinant Cas9 protein were complexed with different amounts of nontargeting IVT gRNA, and RNPs were transfected into HEK293 cells using CRISPRMAX lipofection reagent [27]. We harvested cells 30 h after transfection and measured transcript levels of *interferon beta 1* (*IFNB1)* and *ISG15* by quantitative real-time PCR (qRT-PCR; **Fig 1A**). Introduction of gRNAs caused a dramatic increase in both *IFNB1* and *ISG15* levels, and the presence of Cas9 protein did not have an effect on the outcome. Cas9 on its own did not induce *IFNB1* or *ISG15* expression. To our surprise, as little as 1 nM of gRNA was sufficient to trigger a 30–50-fold increase in the transcription of innate immune genes. We further found that a commonly administered amount of 50 nM gRNA can induce *IFNB1* by 1,000-fold, which is equal to induction by canonical IFNβ inducers such as viral mRNA from Sendai virus [28] or a hepatitis C virus (HCV) PAMP [21,29] (**Fig 1B**).

RNPs can be delivered into cells via different transfection methods, and while lipofection is cost-effective and easy to use, many researchers prefer electroporation for harder-to-transfect cells. We wondered if the transfection method would affect the IFNβ response and compared gRNA transfection via lipofection (Lipofectamine 2000 and RNAiMAX) to nucleofection (Lonza) (**Fig 1C**). Lipofection led to a strong increase in *IFNB1* and *ISG15* transcript levels after as little as 6 h posttransfection, and the response was sustained for up to 48 h. Nucleofection also caused an increase in innate immune signaling at early time points, but the response was milder than in lipofected samples and was greatly diminished by 48 h.

**Fig 2:**
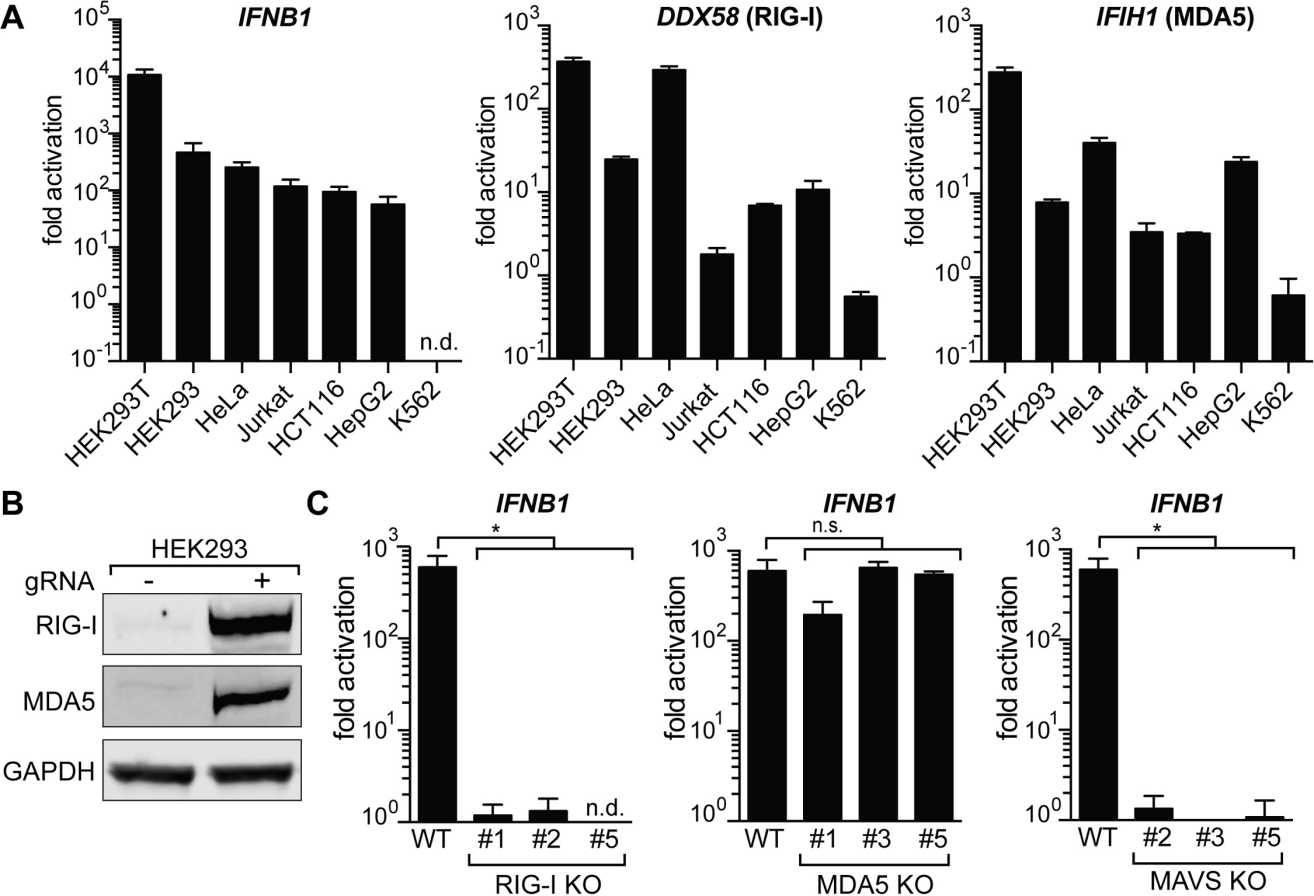
IVT gRNAs are recognized via the RIG-I pathway. (A) qRT-PCR analysis of increase in *IFNB1* transcript levels (left) and transcript levels of the two main cytosolic RIG-I-like receptors (*DDX58* and *IFIH1*) after introduction of IVT gRNA. Cell lines were ordered by responsiveness to gRNA-mediated induction of *IFNB1* transcript levels. Cells were harvested for RNA extraction 30 h after transfection. Ct values were normalized to Ct values of mock-transfected cells for each cell line to determine fold activation. *IFNB1* levels for K562 cells were too low to be determined (n.d.). (B) Western blot analysis for RIG-I and MDA5 expression of mock-transfected and gRNA-transfected HEK293 cells after 48 h. (C) qRT-PCR analysis of *IFNB1* transcript levels in HEK293 RIG-I (left), MDA5 (middle), and MAVS (right) KO cells. Shown are three biological replicates of three clonal populations of RIG-I, MDA5, or MAVS KO cells, respectively. *IFNB1* levels for RIG-I KO clone #5 were too low to be determined (n.d.). For panels A and C, cells were harvested for RNA extraction 30 h after transfection using RNAiMAX transfection reagent. Average values of three biological replicates +/−SD are shown. Statistical significances were calculated by unpaired *t* test (*p < 0.0001). The underlying data for this figure can be found in S1 Data. Ct, cycle threshold; *DDX58*, *DExD-H-box helicase 58*; GAPDH, glyceraldehyde 3-phosphate dehydrogenase; gRNA, guide RNA; HEK293, human embryonic kidney 293; HEK293T, human embryonic kidney cells 293 SV40 large T antigen; *IFIH1*, *interferon induced with helicase C domain 1*; *IFNB1*, *interferon beta 1*; IVT, in vitro–transcribed; KO, knockout; MAVS, mitochondrial antiviral signaling; MDA5, melanoma differentiation–associated gene 5; n.d., not determined; n.s., not significant; qRT-PCR, quantitative real-time PCR; RIG-I, retinoic acid–inducible gene I; WT, wild type.

Next, we asked if the innate immune response to gRNAs is a common phenomenon across different cell types and compared IFNβ activation in seven commonly used human cell lines of various lineages: human embryonic kidney cells 293 SV40 large T antigen (HEK293T), HEK293, Henrietta Lacks cells (HeLa), Jurkat, HCT116, HepG2, and K562 (**Fig 2A**). While the magnitude of induction varied between cell lines, all tested cell lines responded to IVT gRNA transfection with activation of *IFNB1* expression. The sole exception was K562 cells, which have a homozygous deletion of the *IFNA* and *IFNB1* genes [30]. We also measured transcript levels of two major cytosolic pathogen recognition receptors, RIG-I (*DExD-H-box helicase 58* [*DDX58*]) and MDA5 (*interferon induced with helicase C domain 1* [*IFIH1*]), and noticed that all cell lines except K562 up-regulated these transcripts in response to introduction of gRNAs. We also confirmed these results on the protein level in HEK293 cells (**Fig 2B**).

The RIG-I and MDA5 receptors complement each other by recognizing different structures in foreign cytosolic RNAs, but the exact nature of their ligands is not yet fully understood [31,32]. To investigate if IVT gRNAs are recognized via RIG-I or MDA5, we generated clonal knockout (KO) cell lines for RIG-I, MDA5, and their downstream interaction partner MAVS in HEK293 cells using CRISPR-Cas9. As the expressions of both RIG-I and MDA5 are themselves stimulated by IFNβ, we confirmed successful KO after transfection with gRNAs by genomic PCR, Sanger sequencing, and western blot (**S1A–S1C Fig**). MAVS KO cells were confirmed by western blot (**S1D Fig**). Strikingly, activation of *IFNB1* expression after introduction of gRNAs was absent in RIG-I and MAVS KO cells, while MDA5 KO cells did not show a significant decrease in *IFNB1* transcript levels (**Fig 2C**). This indicates that IVT gRNAs are exclusively recognized through RIG-I to trigger a type I interferon response.

**Fig 3:**
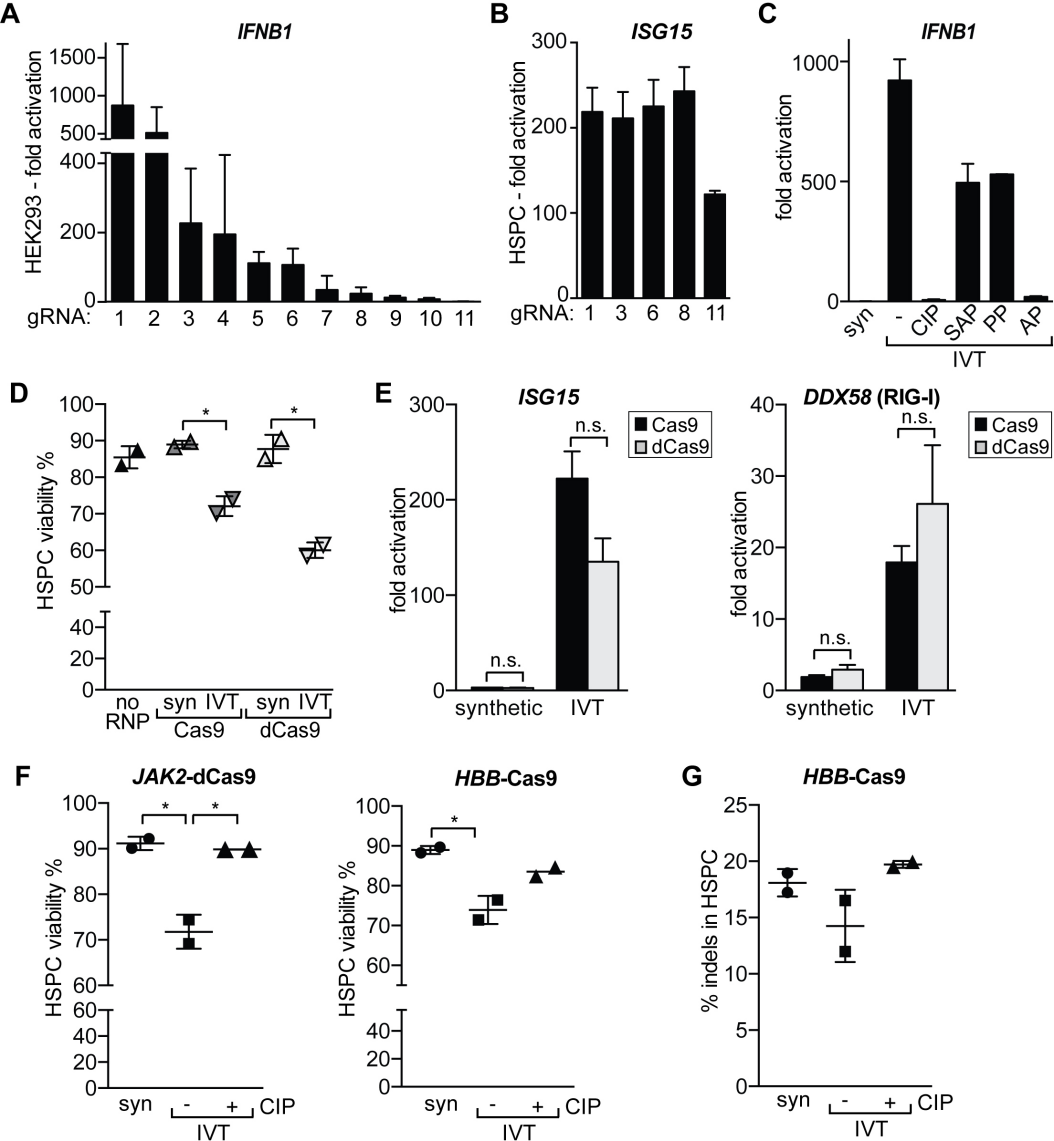
Protospacer and 5’-triphosphate determine the intensity of the gRNA-mediated IFNβ response. (A) qRT-PCR analysis of *IFNB1* transcript levels in HEK293 cells transfected with equal amounts of gRNAs containing different 20-nucleotide protospacers. gRNAs were ordered by decreasing levels of *IFNB1* activation. gRNA1 refers to the gRNA that has been used in all previous experiments. (B) qRT-PCR analysis of *ISG15* transcript levels in primary HSPCs nucleofected with equal amounts of gRNA 1, 3, 6, 8, and 11 from panel A. Average values of two biological replicates +/−SD are shown. (C) qRT-PCR analysis of *IFNB1* transcript levels in HEK293 cells transfected with synthetic (“syn”), IVT, and phosphatase-treated IVT gRNAs (gRNA1). (D) Viability of human primary HSPCs 24 h postnucleofection with no RNP and Cas9 or dCas9 RNPs. dCas9 or Cas9 were complexed with synthetic (“syn”) or IVT gRNA targeting the *HBB* gene. Viability was determined by trypan blue exclusion test. (E) qRT-PCR analysis of *ISG15* and *DDX58* (RIG-I) transcript levels in human primary HSPCs 16 h postnucleofection. dCas9 or Cas9 were complexed with synthetic or IVT gRNA targeting the *HBB* gene, respectively. Ct values were normalized against Ct of mock-nucleofected cells. Average values of two biological replicates +/−SD are shown. (F) Viability of human primary HSPCs 16 h posttransfection with RNPs. RNPs consisted of dCas9 complexed with synthetic, IVT, or CIP-treated IVT gRNAs targeting a noncoding intron of *JAK2* (left panel) or Cas9 complexed with gRNAs targeting exon 1 of *HBB* (right panel). Viability was determined by trypan blue exclusion test. (G) Editing outcomes in HSPCs 48 h after nucleofection with RNPs targeting the *HBB* locus. Indel frequencies were determined by amplicon NGS. Statistical significances were calculated by unpaired *t* test (*p < 0.05, **p < 0.01, ***p < 0.0001). The underlying data for this figure can be found in S1 Data. AP, thermosensitive alkaline phosphatase; Cas9, CRISPR-associated 9; CIP, calf intestinal alkaline phosphatase; Ct, cycle threshold; dCas9, nuclease-dead Cas9; *DDX58*, *DExD-H-box helicase 58*; gRNA, guide RNA; HEK293, human embryonic kidney 293; HSPC, CD34^+^ human hematopoietic stem and progenitor cell; *HBB*, *hemoglobin subunit beta*; IFNβ, interferon beta; *IFNB1*, *interferon beta 1*; indel, insertion and deletion; IVT, in vitro–transcribed; *JAK2*, *Janus kinase 2*; NGS, next-generation sequencing; n.s., not significant; qRT-PCR, quantitative real-time PCR; PP, 5’ RNA polyphosphatase; RIG-I retinoic acid–inducible gene I; RNP, ribonucleoprotein; SAP, shrimp alkaline phosphatase.

As the structural requirements of RIG-I ligands are still not completely understood, we wondered if different 20-nucleotide protospacers in gRNAs vary in their potency to trigger an innate immune response via RIG-I. We designed 10 additional nontargeting gRNAs that we in vitro transcribed and transfected into HEK293 cells. Surprisingly, we found that the cells responded to different protospacers with a wide range of *IFNB1* expression. Several gRNAs produced very little innate immune response, and one gRNA (gRNA11) yielded no *IFNB1* activation at all (**Fig 3A**). We speculated that the differential response may be correlated with the purity of the RNA product after in vitro transcription or the stability of the secondary structure of the RNA [33,34]. However, we found that there was no obvious correlation between the immune response to certain gRNAs and their purity; predicted protospacer secondary structure; full secondary structure, including the constant region; or predicted disruption of the constant region by mispairing with the protospacer (**S2 Fig**). When we separately nucleofected five of these gRNAs into primary CD34^+^ human hematopoietic stem and progenitor cells (HSPCs), we found that all gRNAs induced a strong immune response. Only gRNA11, which showed no immune stimulation in HEK293 cells, resulted in half the amount of *ISG15* transcript (**Fig 3B**). These results indicate that RIG-I recognition patterns of IVT gRNAs are complex and difficult to anticipate a priori based on predicted properties of the variable protospacer and cell type.

One well-established structural requirement of RIG-I ligands is the presence of a 5’-triphosphate group [35]. We asked if preparations that remove the 5’ triphosphate might avoid or reduce the innate immune response to IVT gRNAs. We first used a synthetic gRNA that lacks a 5’-triphosphate and verified that this gRNA does not induce *IFNB1* expression when transfected into HEK293 cells (**Fig 3C**). Synthetic gRNAs are becoming more commonplace but are still an order of magnitude more expensive than in vitro transcription of gRNAs. This limits their application for high-throughput interrogation of gene function in primary cells. We therefore asked if treatment of IVT gRNA with phosphatases that remove the 5’-triphosphate would reduce *IFNB1* induction. We tested calf intestinal alkaline phosphatase (CIP), shrimp alkaline phosphatase (SAP), 5’-RNA polyphosphatase (PP), and thermosensitive alkaline phosphatase (AP) and found that phosphatase treatment with CIP and AP abolished the *IFNB1* response, while SAP and PP treatment only resulted in a reduction of the response (**Fig 3C**). We also compared purification of IVT gRNAs by solid-phase reversible immobilization (SPRI) beads to column purification and established that SPRI bead cleanup is not sufficient to completely avoid an immune response, even when more phosphatase is used (**S3A–S3B Fig**). Taken together, these results indicate that 5’-triphosphate is a necessary requirement for gRNA-induced *IFNB1* activation through RIG-I but that additional structural properties of the gRNAs also influence the magnitude of the immune response.

Next, we asked if phosphatase treatment alters the genome editing potential of gRNAs. As gRNA1 targets the BFP gene, we used a HEK293T cell line with a stably integrated BFP reporter [26], nucleofected cells with phosphatase-treated gRNA-Cas9 RNPs, and monitored editing outcomes by T7 endonuclease I assay (**S3C Fig**). We did not observe any significant difference in editing outcomes between synthetic, IVT, and phosphatase-treated gRNAs, suggesting that phosphatase treatment does not affect the function of the gRNA.

When a cell initiates an antiviral immune response, it also undergoes cellular stress that can affect cell viability [36,37]. Hence, we asked if there is a correlation between the IFNβ response and cell viability after transfection with synthetic, IVT, or CIP-treated IVT gRNA. Not surprisingly, the viability of the very robust HEK293 cell line was not affected by the antiviral immune response (**S3D Fig**). We then turned to HSPCs, which are a much more sensitive cell type. We first nucleofected HSPCs with RNPs targeting the *hemoglobin subunit beta* (*HBB*) gene [12] and compared synthetic and IVT gRNA interferon stimulation and cell viability posttransfection. Double-strand breaks (DSBs) have been reported to cause innate immune stimulation and can themselves cause decreases in cell fitness [38,39]. Therefore, we performed controls using nuclease-dead Cas9 (dCas9) to form RNPs and confirmed by Sanger sequencing and TIDE analysis that dCas9-RNPs did not induce DSBs [40] (**S3E Fig**).

We found a significant decrease in HSPC viability using both of the IVT gRNA RNPs that had an increase in IFN-stimulated genes *ISG15* and *RIG-I* (**Fig D-E**). We did not see a substantial difference in viability or ISG expression between Cas9 and dCas9 RNPs, suggesting that nuclease activity leading to DNA damage did not cause the immune response. Next, we asked if CIP treatment of gRNAs could reverse the decrease in viability in HSPCs. We nucleofected HSPCs with dCas9 RNPs targeting a noncoding intron of *Janus kinase 2* (*JAK2*) or Cas9 RNPs targeting the *HBB* gene and compared synthetic, IVT, and CIP-treated IVT gRNAs. Strikingly, CIP treatment restored viability in HSPCs (**Fig 3F**). We were also interested in editing outcomes in these samples and performed amplicon next-generation sequencing (NGS) for the *HBB* locus. While the phosphatase-treated gRNA performed similarly to the synthetic gRNA, the IVT gRNA resulted in slightly fewer insertions and deletions (indels) (**Fig 3G**).

## Discussion

**Fig 4:**
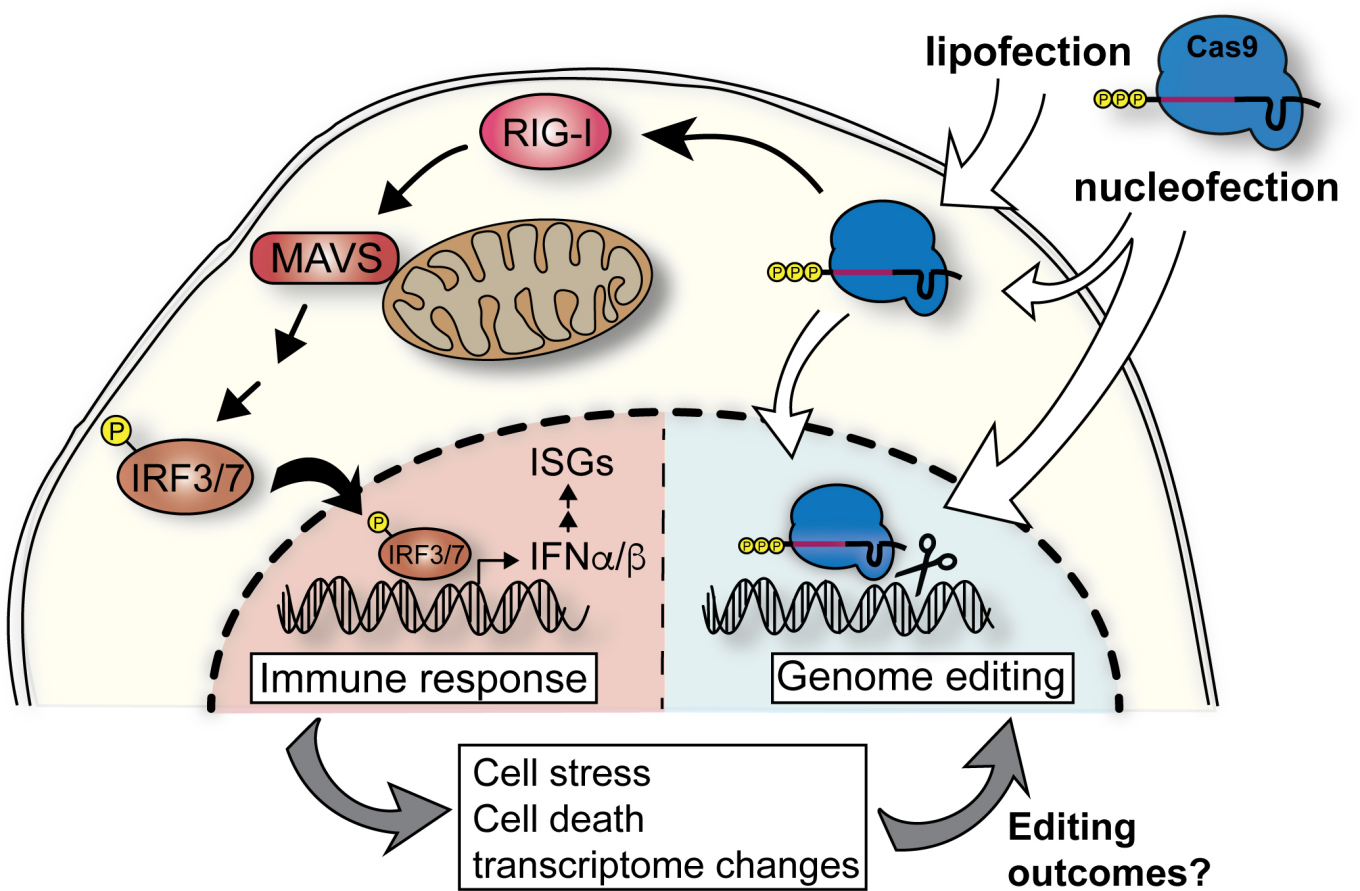
Transfection of IVT gRNAs induces a cytosolic immune response. Proposed model of IVT gRNA recognition pathways in mammalian cells. IVT gRNAs carry a 5’-triphosphate, and when complexed with Cas9 protein and transfected into cells, cytosolic RNPs are recognized by RIG-I, triggering a cascade of activation events through the MAVS. This results in phosphorylation of IRF3/7 and their shuttling into the nucleus to activate expression of type I interferons (IFNα/β). This triggers the expression of ISGs. This innate immune response changes the transcriptome of the cell and can cause cell stress and/or death, which in turn might affect the editing outcomes. Cas9, CRISPR-associated 9; gRNA, guide RNA; IFNα/β, interferon alpha/beta; IRF3/7, interferon regulatory factor 3/7; ISG, interferon-stimulated gene; IVT, in vitro–transcribed; MAVS, mitochondrial antiviral signaling protein; RIG-I, retinoic acid–inducible gene I; RNP, ribonucleoprotein.

We have found that IVT gRNAs used with Cas9 RNPs for many genome-editing experiments can trigger a strong innate immune response in many mammalian cell types (**Fig 4**). Lipofection results in a stronger and longer-lasting response than nucleofection, possibly because lipofection delivers gRNAs to the cytosol, while nucleofection delivers mainly to the nucleus. Using isogenic KO clones, we found the gRNA-induced response is mediated via the antiviral RIG-I pathway and results in expression of genes that initiate an antiviral immune response. While introduction of IFN-stimulating gRNAs does not affect viability in HEK293 cells, we found that viability of primary HSPCs is negatively affected by the antiviral immune response. While DSBs have on their own been reported to induce an innate immune response [38], we found triphosphate-containing gRNAs complexed with dCas9 induce an immune response and cell death in HSPCs. Only removal of the triphosphate is sufficient to reduce gRNA-induced innate immune signaling.

These results have several implications. We suggest that the gene signature associated with type I interferon stimulation should be considered when studying the transcriptome of recently edited bulk populations of cells. Furthermore, all mammalian cells can both produce type I interferons and also respond to them through the ubiquitously expressed receptor interferon alpha and beta receptor subunit 1 (IFNAR1) [41]. Even cells that have not been successfully transfected with RNPs could sense the IFNβ produced by neighboring cells and activate downstream antiviral defense mechanisms. This could be an important consideration during *in vivo* genome editing applications, as RNP delivery into one set of cells could provoke a widespread innate immune response in the surrounding tissues.

We found that synthetic gRNAs completely circumvent the RIG-I mediated response, offering a valuable path to avoid innate immune signaling during therapeutic editing. However, synthetic gRNAs can become expensive when performing experiments that require testing or using many gRNAs. We found that a cost-effective phosphatase treatment to remove the 5’-triphosphate before transfection reduces the immune response and increases posttransfection viability in HSPCs. Furthermore, editing outcomes in cell lines with phosphatase-treated gRNA were comparable to those of IVT gRNAs, suggesting that removal of 5’-phosphate groups does not abolish gRNA function. In fact, in sensitive HSPCs, phosphatase-treated gRNA slightly outperformed IVT gRNA, which is possibly due to reduced viability in samples transfected with IVT-RNPs. Thus, consideration of a potential innate immune stimulation prior to choice of genome editing reagents, study design, and implementation of controls is critical when performing genome editing using RNPs in mammalian cells.

While we were preparing this manuscript for submission, the Kim group reported similar results in HeLa cells and primary human CD4^+^ T cells [42]. They confirmed that the type I interferon response is dependent on the presence of a 5’-triphopsphate group and that CIP treatment can increase viability by avoiding the antiviral response. These results are very much in alignment with our findings and extend the potential problem of innate immune signaling to additional cell types.

Our study adds extra depth by further outlining the mechanisms by which gRNAs are sensed. We show that gRNA sensing depends upon RIG-I and MAVS, but MDA5 KO cells are fully capable of inducing IFNβ after IVT gRNA transfection. Hence, gRNA sensing is independent of the MDA5 PAMP receptor, consistent with RIG-I’s preference for short double-stranded RNA (dsRNA) structures and MDA5’s preference for long dsRNA fragments [43]. Furthermore, we show that in addition to a 5’-triphosphate, the protospacer sequence is also critical to determine the intensity of the IFNβ response. Not only do different gRNAs induce different innate immune responses, but some gRNAs induce no response at all. However, this seems to be cell-type specific, as we found that sensitive cells such as primary HSPCs react to the same gRNAs with a strong immune response independently of the protospacer. It has been proposed that 5’-base-paired RNA structures are required to activate antiviral signaling via RIG-I, but we found no correlation between signaling and a variety of predicted RNA properties, including secondary structure [33]. Our results therefore suggest that the mechanism of gRNA sensing by the RIG-I pathway is relatively complex in that it requires 5’-triphosphates but that this moiety is not sufficient to induce the response. Additionally, we have not ruled out the possibility that gRNAs could be recognized by Toll-like receptors (TLRs), though we and others [42] have found that KO of RIG-I is sufficient to completely abrogate gRNA-induced signaling in multiple cell contexts. The role of TLR recognition could be addressed in future work to delineate the full set of molecular features responsible for gRNA activation of innate immunity, which might yield accurate predictors of innate immune signaling in general.

## Materials and methods

### In vitro transcription of gRNAs

gRNA was synthesized by assembly PCR and in vitro transcription as previously described [12]. Briefly, a T7 RNA polymerase substrate template was assembled by PCR from a variable 58–59 nt primer containing T7 promoter, variable gRNA guide sequence, the first 15 nt of the nonvariable region of the gRNA (T7FwdVar primers, 10 nM, S1 and S2 Tables for gRNA sequences), and an 83 nt primer containing the reverse complement of the invariant region of the gRNA (T7RevLong, 10 nM), along with amplification primers (T7FwdAmp, T7RevAmp, 200 nM each). The two long primers anneal in the first cycle of PCR and are then amplified in subsequent cycles. Phusion high-fidelity DNA polymerase was used for assembly (New England Biolabs). Assembled template was used without purification as a substrate for in vitro transcription by T7 RNA polymerase, using the HiScribe T7 High Yield RNA Synthesis kit (New England Biolabs) following the manufacturer’s instructions. Resulting transcription reactions were treated with DNAse I (New England Biolabs), and RNA was purified by treatment with a 5X volume of homemade SPRI beads (comparable to Beckman-Coulter AMPure beads) and elution in RNAse-free water.

### Phosphatase treatment of IVT gRNAs

gRNAs were treated with phosphatases as follows: CIP (New England Biolabs, 30 U), SAP (New England Biolabs 10 U), PP (Lucigen, 20 U), and FastAP AP (Thermo Fisher Scientific, 10 U) were added per 20 μl in vitro transcription reaction, and samples were incubated at 37°C for 3 h before proceeding to purification and DNAseI treatment. gRNA was purified using a Qiagen RNeasy Mini Kit (Qiagen) or by 5X volume of homemade SPRI beads (comparable to Beckman-Coulter AMPure beads). The detailed protocol and additional notes are available online (dx.doi.org/10.17504/protocols.io.nghdbt6).

### In vitro transcription of HCV PAMP and Sendai virus DI RNA

HCV PAMP in vitro transcription template [21] was generated by annealing HCV fwd and rev (5 μM each) oligos (S1 Table). In the subsequent in vitro transcription reaction, 2 μl of the annealed product was used as DNA template, using HiScribe T7 High Yield RNA Synthesis kit (New England Biolabs).

The plasmid containing the SeV DI RNA[28] was a gift from Prof. Peter Palese, Icahn School of Medicine at Mount Sinai, New York. Plasmid was digested with HindII/EcoRI before in vitro transcription with HiScribe T7 High Yield RNA Synthesis kit (New England Biolabs). The sequence of the IVT DI, including the T7 promoter, hepatitis delta virus ribozyme, and the T7 terminator, is TAATACGACTCACTATA**ACCAGACAAGAGTTTAAGAGATATGTATCCTTTTAAATTTTCTTGTCTTCTTGTAAGTTTTTCTTACTATTGTCATATGGATAAGTCCAAGACTTCCAGGTACCGCGGAGCTTCGATCGTTCTGCACGATAGGGACTAATTATTACGAGCTGTCATATGGCTCGATATCACCCAGTGATCCATCATCAATCACGGTCGTGTATTCATTTTGCCTGGCCCCGAACATCTTGACTGCCCCTAAAATCTTCATCAAAATCTTTATTTCTTTGGTGAGGAATCTATACGTTATACTATGTATAATATCCTCAAACCTGTCTAATAAAGTTTTTGTGATAACCCTCAGGTTCCTGATTTCACGGGATGATAATGAAACTATTCCCAATTGAAGTCTTGCTTCAAACTTCTGGTCAGGGAATGACCCAGTTACCAATCTTGTGGACATAGATAAAGATAGTCTTGGACTTATCCATATGACAATAGTAAGAAAAACTTACAAGAAGACAAGAAAATTTAAAAGGATACATATCTCTTAAACTCTTGTCTGGT**GGCCGGCATGGTCCCAGCCTCCTCGCTGGCGCCGGCTGGGCAACATTCCGAGGGGACCGTCCCCTCGGTAATGGCGAATAGCATAACCCCTTGGGGCCTCTAAACGGGTCTTGAGGGGTTTTTTG.

The sequence of the SeV DI is highlighted in boldface.

Both HCV PAMP and SeV DI RNA were purified by treatment with a 5X volume of homemade SPRI beads (comparable to Beckman-Coulter AMPure beads) and elution in RNAse-free water.

### Synthetic gRNAs

Chemically synthesized gRNAs, which were purified using high-performance liquid chromatography (HPLC), were purchased from Synthego.

### RNA quality control

IVT gRNAs were analyzed using a Bioanalyzer. This was performed by the UC Berkeley Functional Genomics Laboratory (FGL) core facility. gRNAs were denatured for 5 min at 70°C before analysis on bioanalyzer.

### Cas9 protein preparation

The Cas9 construct (pMJ915) contained an N-terminal hexahistidine-maltose binding protein (His6-MBP) tag, followed by a peptide sequence containing a tobacco etch virus (TEV) protease cleavage site. The protein was expressed in *Escherichia coli* strain BL21 Rosetta 2 (DE3; EMD Biosciences) grown in TB medium at 16°C for 16 h following induction with 0.5 mM IPTG. The Cas9 protein was purified by a combination of affinity, ion exchange, and size exclusion chromatographic steps. Briefly, cells were lysed in 20 mM HEPES pH 7.5, 1 M KCl, 10 mM imidazole, 1 mM TCEP, 10% glycerol (supplemented with protease inhibitor cocktail [Roche]) in a homogenizer (Avestin). Clarified lysate was bound to Ni-NTA agarose (Qiagen). The resin was washed extensively with lysis buffer, and the bound protein was eluted in 20 mM HEPES pH 7.5, 100 mM KCl, 300 mM imidazole, 1 mM TCEP, 10% glycerol. The His6-MBP affinity tag was removed by cleavage with TEV protease, while the protein was dialyzed overnight against 20 mM HEPES pH 7.5, 300 mM KCl, 1 mM TCEP, 10% glycerol. The cleaved Cas9 protein was separated from the fusion tag by purification on a 5 ml SP Sepharose HiTrap column (GE Life Sciences), eluting with a linear gradient of 100 mM–1 M KCl. The protein was further purified by size exclusion chromatography on a Superdex 200 16/60 column in 20 mM HEPES pH 7.5, 150 mM KCl, and 1 mM TCEP. Eluted protein was concentrated to 40 uM, flash-frozen in liquid nitrogen, and stored at −80°C.

### Culture and transfection of immortalized cell lines

Cells were obtained from ATCC and verified mycoplasma-free (Mycoalert LT-07, Lonza). HEK293, HEK293T, HCT116, HepG2, and HeLa cells were maintained in DMEM supplemented with 10% FBS and 100 μg/mL penicillin-streptomycin (all Gibco). K562 and Jurkat cells were maintained in RPMI supplemented with 10% FBS and 100 μg/mL penicillin-streptomycin.

All transfections in cell lines were performed in 12-well cell culture dishes using 2 × 10^5^ cells per transfection. For lipofection, we used Lipofectamine CRISPRMAX-Cas9, Lipofectamine RNAiMAX, or Lipofectamine 2000 Transfection Reagent (all Invitrogen) in reverse transfections according to the manufacturer’s protocols. Unless stated otherwise, 2 × 10^5^ cells were transfected with 50 pmol of RNA to a final concentration of 50 nM and harvested 24–30 h posttransfection for RNA extraction.

### Culture and transfection of primary HSPCs

HSPCs from mobilized peripheral blood (Allcells) were thawed and cultured in StemSpan SFEM medium (StemCell Technologies) supplemented with StemSpan CC110 cocktail (StemCell Technologies) for 48 h before nucleofection with dCas9 or Cas9 RNP (75 pmol of dCas9, 75 pmol of gRNA). Then, 1.5 × 10^5^ HSPCs were pelleted (100 × g, 10 min) and resuspended in 20 μl Lonza P3 solution, mixed with 10 μl dCas9 or Cas9 RNP, and nucleofected using ER100 protocol in Lonza 4D nucleofector. Viability of the cells was measured 24 h postnucleofection using trypan blue exclusion test. RNA was harvested 16 h postnucleofection.

### RNA extraction, cDNA synthesis, and qRT-PCR

Cell cultures were washed with PBS prior to RNA extraction. Total RNA was extracted using RNeasy Miniprep columns (Qiagen) according to the manufacturer’s instructions, including the on-column DNAseI treatment (Qiagen). One μg of total RNA was used for subsequent cDNA synthesis using Reverse Transcription Supermix (Biorad). For qRT-PCR reactions, a total of 20 ng of cDNA was used as a template and combined with primers (see S3 Table), and EvaGreen Supermix (Biorad) and amplicons were generated using standard PCR amplification protocols for 40 cycles on a StepOnePlus Real-Time PCR system (Applied Biosystems). Ct values for each target gene were normalized against Ct values obtained for *GAPDH* to account for differences in loading (ΔCt). To determine “fold activation” of genes, ΔCt values for target genes were then normalized against ΔCt values for the same target gene for mock-treated cells (ΔΔCt).

### Generation of KO cell lines

For CRISPR/Cas9 genome editing, we used a plasmid encoding both the Cas9 protein and the gRNA. pSpCas9(BB)-2A-GFP (px458) was a gift from Feng Zhang (Addgene plasmid #48138). We designed gRNA sequences using the free CRISPR KO design online tool from Synthego. Two different gRNA sequences were designed for RIG-I and MDA5, respectively (see S3 Table).

Using a Lonza 4D nucleofector (Lonza) with the manufacturer’s recommended settings, 2 × 10^5^ HEK293 cells were nucleofected with 2 μg of px458 plasmids containing both targeting gRNAs in a 1:1 ratio. After 48 h, cells were harvested and subjected to fluorescence-activated cell sorting (FACS). Cells expressing high levels of GFP were single-cell sorted into 96-well plates to establish clonal populations.

For the screening process, genomic DNA (gDNA) from clonal populations was extracted using QuickExtract solution (Lucigen). For KO of RIG-I and MDA5, we screened clones by genomic PCR, looking for a PCR product that is significantly smaller in size than that of WT HEK293 cells (see S4 Table for primers). PCR products were then Sanger sequenced by the UC Berkeley DNA Sequencing facility using the forward primers of the PCR reaction as sequencing primers.

### Western blot

Cells were harvested and washed with PBS. Cells were lysed in 1x RIPA buffer (EMD Millipore) for 10 min on ice. Samples were spun down at 14,000 × g for 15 min, and protein lysates were transferred to a new tube. Fifty μg of total protein was separated for size by SDS-PAGE and transferred to a nitrocellulose membrane. Blots were blocked in 4% skim milk in 50 mm Tris-

HCl (pH 7.4), 150 mm NaCl, and 0.05% Tween 20 (TBST) and then probed for RIG-I, MDA5, MAVS, or GAPDH protein using antibodies against RIG-I (D14G6), MDA5 (D74E4), MAVS (D5A9E), or GAPDH (14C10), respectively (all Cell Signaling Technologies). This was followed by incubation with secondary antibody IRDye 800CW Donkey anti-Rabbit IgG (Li-Cor). Protein standards (GE Healthcare) were loaded in each gel for size estimation. Blots were visualized using a Li-Cor Odyssey Clx (Li-Cor).

### T7 endonuclease I assay

Cells were harvested 24 h after transfection and washed with PBS. gDNA was extracted using QuickExtract solution (Lucigen) following the manufacturer’s protocol. PCR across the target site in the BFP gene was run using the BFP amplicon primer set (S4 Table). Two hundred ng of PCR product was heated to 100°C and slowly cooled down to let DNA reanneal. Annealed DNA was digested with T7 endonuclease I (NEB) for 20 min at 37°C. DNA was then analyzed by agarose gel electrophoresis.

### TIDE analysis

PCR products were generated with target-specific HBB primer set 1, sequenced, and Sanger traces were then analyzed with the *TIDE* webtool (http://tide.nki.nl).

### PCR and next-generation amplicon sequencing preparation

Using primer set 1, 50–100 ng of gDNA from edited CD34+ cells was amplified at *HBB* sites (S4 Table). The PCR products were SPRI cleaned, followed by amplification of 20–50 ng of the first PCR product in a second 12-cycle PCR using primer set 2 (S4 Table). Then, the second PCR products were SPRI cleaned, followed by amplification of 20–50 ng of the second PCR product in a third 9 cycle PCR using illumina-compatible primers (primers designed and purchased through the Vincent J. Coates Genomics Sequencing Laboratory [GSL] at University of California, Berkeley), generating indexed amplicons of an appropriate length for NGS. Libraries from 100–500 pools of edited cells were pooled and submitted to the GSL for paired-end 300 cycle processing using a version 3 Illumina MiSeq sequencing kit (Illumina, San Diego, CA) after quantitative PCR measurement to determine molarity.

### Next-generation amplicon sequencing analysis

Samples were deep sequenced on an Illumina MiSeq at 300 bp paired-end reads to a depth of at least 10,000 reads. A modified version of CRISPResso [44] was used to analyze editing outcomes. Briefly, reads were adapter trimmed and then joined before performing a global alignment between reads and the reference sequence using NEEDLE [45]. Indel rates were calculated as any reads in which an insertion or deletion overlaps the cut site or occurs within 3 base pairs of either side of the cut site, divided by the total number of reads.

## Supporting information

S1 Fig Validation of HEK293 RIG-I, MDA5, and MAVS KO cell lines. (A) Genomic PCR analysis of the RIG-I and MDA5 genomic loci, respectively. KO clones showed a PCR product that was substantially different in size compared to WT HEK293 cells. (B) Alignment of Sanger sequencing tracks of PCR products shown in (A) to the WT reference sequence. gRNAs used in the experiment are highlighted on the reference sequence with black boxes; their PAM sequences are shown with red boxes. All three RIG-I clones showed the same 58 bp deletion homozygously. MDA5 KO #1 and #3 had a homozygous 59 bp deletion; clone #5 had the same 59 bp deletion on one allele and a large insertion on the other allele. (C) Western blot analysis for RIG-I and MDA5 expression in HEK293 RIG-I, and MDA5 KO cells. Cells were transfected with 50 nM of gRNA to stimulate an IFNβ response and then harvested for protein extraction after 48 h. (D) Western blot analysis for MAVS expression in HEK293 WT and MAVS KO cells. Our KO strategy targeted the main isoform of MAVS (shown by arrow). Asterisk indicates nonspecific band. gRNA, guide RNA; HEK293, human embryonic kidney 293; IFNβ, interferon beta; KO, knockout; MAVS, mitochondrial antiviral signaling; MDA5, melanoma differentiation–associated gene 5; PAM, protospacer-adjacent motif; RIG-I, retinoic acid–inducible gene I; WT, wild-type.
(TIF)

S2 Fig gRNA purity and stability show no direct correlation to the IFNβ response. (A) Bioanalyzer results for gRNAs tested in Fig 3A. IVT gRNAs were denatured for 5 min at 70°C before analysis. (B) Correlation between *IFNB1* activation and RNA stability or hamming distance, respectively. Predicted RNA secondary structure was calculated using Vienna RNA Fold [46]. Hamming distance reflects the extent to which the protospacer might interact with the gRNA constant region. The predicted secondary structure of the constant region in isolation was compared to the predicted secondary structure of the constant region when paired with the protospacer. The hamming distance between the dot-bracket notation–predicted secondary structure in each context is shown. gRNA, guide RNA; IFNβ, interferon beta; *IFNB1*, *interferon beta 1*; IVT, in vitro–transcribed.
(TIF)

S3 Fig gRNA purification and complete removal of 5’-triphosphate groups are essential to avoid an innate immune response. (A) qRT-PCR analysis of *IFNB1* transcript levels in HEK293 cells transfected with synthetic, IVT, and CIP IVT gRNAs (gRNA1). After in vitro transcription and CIP-treatment, gRNAs were purified with SPRI beads or spin columns, respectively. Cells were harvested for RNA extraction 30 h after transfection with RNAiMAX transfection reagent. Average values of 3 biological replicates +/−SD are shown (B) qRT-PCR analysis of *IFNB1* transcript levels in HEK293 cells transfected with IVT gRNA via RNAiMAX lipofection. IVT gRNAs were treated with 0, 10, 20, or 30 units (U) of CIP, respectively, before purification with SPRI beads. (C) T7E1 assay to determine cleavage efficiencies of phosphatase-treated IVT gRNA-RNPs targeting the *BFP* locus in HEK293T-BFP cells. HEK293T-BFP cells were nucleofected with Cas9/dCas9-RNPs and harvested after 24 h. PCR-amplified target DNA was heated, reannealed, and digested with T7E1 before gel electrophoresis. (D) Viability of HEK293 cells after transfection with gRNAs. Viability was determined using trypan blue exclusion test. (E) Editing outcome in primary HSPCs that were nucleofected with dCas9 or Cas9-IVT gRNA RNPs targeting the *HBB* locus. Amounts of indels were determined 24 h after transfection by PCR across the target site, followed by Sanger sequencing and TIDE analysis. Statistical significances were calculated by unpaired *t* test (*p < 0.05, ***p < 0.0001). The underlying data for this figure can be found in S1 Data. BFP, blue fluorescent protein; Cas9, CRISPR-associated 9; CIP, Calf intestine phosphatase; dCas9, nuclease-dead CRISPR-associated 9; gRNA, guide RNA; HEK293, human embryonic kidney 293; *HBB*, *hemoglobin subunit beta*; *IFNB1*, *interferon beta 1*; indel, insertion and deletion; IVT, in vitro–transcribed; n.s., not significant; qRT-PCR, quantitative real-time PCR; SPRI, solid-phase reversible immobilization; RNP, ribonucleoprotein; T7E1, T7 endonuclease 1.
(TIF)

S1 Table Primers for in vitro transcription. (DOCX)

S2 Table gRNA sequences. gRNA, guide RNA.
(DOCX)

S3 Table qRT-PCR primers. qRT-PCR, quantitative real-time PCR.
(DOCX)

S4 Table genomic PCR primers. (DOCX)

S1 Data Numeric values of all data. (XLSX)

## Abbreviations

AP: thermosensitive alkaline phosphatase
BFP: blue fluorescent protein
Cas: CRISPR-associated
CIP: calf intestinal alkaline phosphatase
CRISPR: clustered, regularly interspaced, short palindromic repeat
crRNA: CRISPR RNA
dCas9: nuclease-dead Cas9
DDX5: DExD-H-box helicase 58
DSB: double-strand break
dsRNA: double-stranded RNA
FACS: fluorescence-activated cell sorting
FGL: Functional Genomics Laboratory
gDNA: genomic DNA
gRNA: guide RNA
GSL: Genomics Sequencing Laboratory
HBB: hemoglobin subunit beta
HCV: hepatitis C virus
HEK293: human embryonic kidney 293
HEK293T: human embryonic kidney cells 293 SV40 large T antigen
HeLa: Henrietta Lacks
His6-MBP: hexahistidine-maltose binding protein
HPLC: high-performance liquid chromatography
HSPC: CD34^+^ human hematopoietic stem and progenitor cell
IFIH1: interferon induced with helicase C domain 1
IFNAR1: interferon alpha and beta receptor subunit 1
IFNB1: interferon beta 1
IFN: interferon beta
Indel: insertion and deletion
IRF3/7: interferon regulatory factor 3/7
ISG: interferon-stimulated gene
ISG15: interferon-stimulated gene 15
IVT: in vitro–transcribed
JAK2: Janus kinase 2
KO: knockout
MAVS: mitochondrial antiviral signaling
MDA5: melanoma differentiation–associated gene 5
n.d: not determined
NGS: next-generation sequencing
n.s.: not significant
PAMP: pathogen-associated molecular pattern
PP: 5’ RNA polyphosphatase
qRT-PCR: quantitative real-time PCR
RIG-I: retinoic acid–inducible gene I
RNP: ribonucleoprotein
SAP: shrimp alkaline phosphatase
SPRI: solid-phase reversible immobilization
TEV: tobacco etch virus
TLR: Toll-like receptor
tracrRNA: *trans*-activating CRISPR RNA
