# Supplementary figures and images for: In vitro–transcribed guide RNAs trigger an innate immune response via the RIG-I pathway
Source: PLoS Biol. 2018 Jul 16;16(7):e2005840. doi: 10.1371/journal.pbio.2005840 (PMC6049001; doi:10.1371/journal.pbio.2005840)

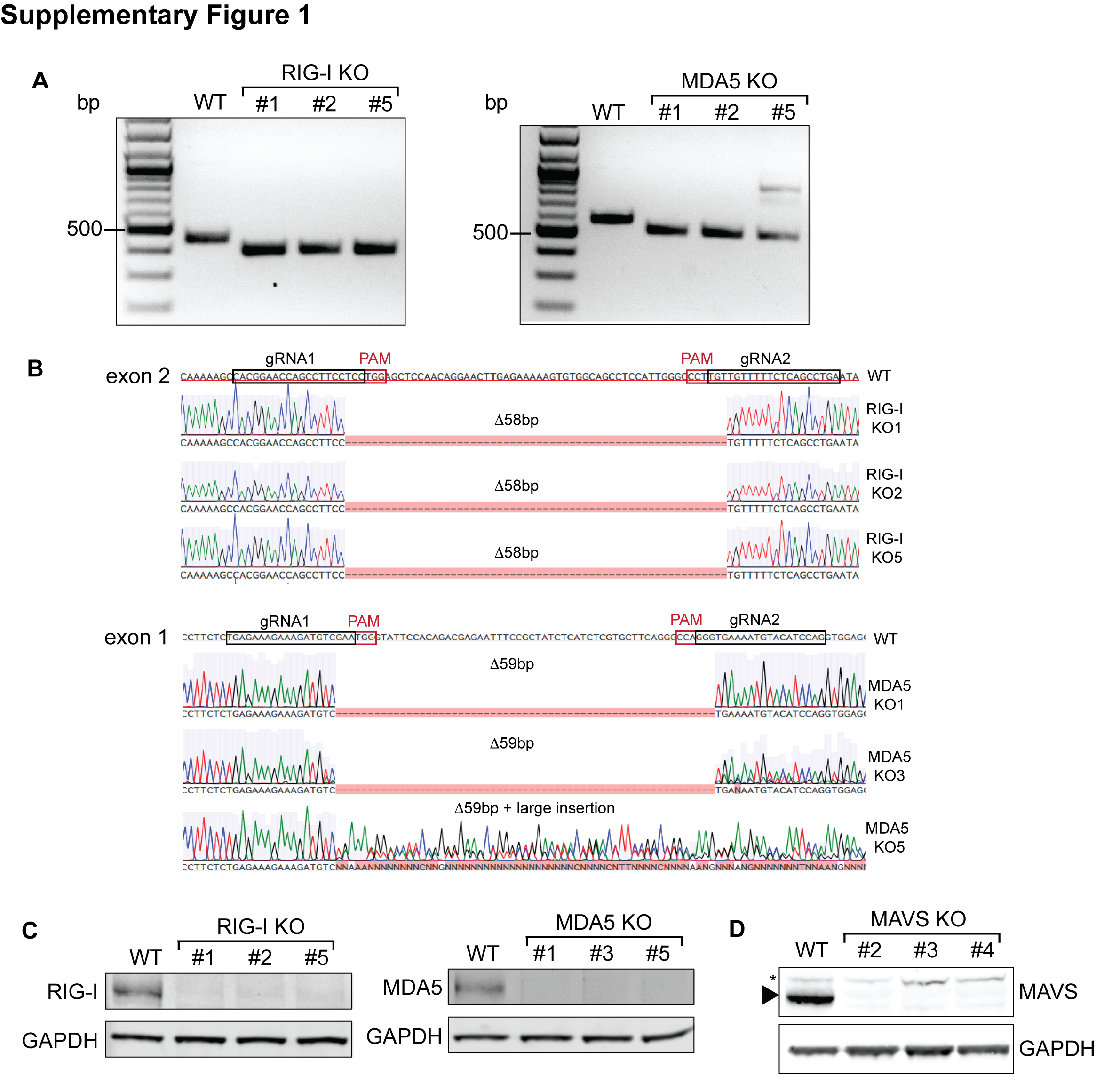

Supplement: S1 Fig — (A) Genomic PCR analysis of the RIG-I and MDA5 genomic loci, respectively. KO clones showed a PCR product that was substantially different in size compared to WT HEK293 cells. (B) Alignment of Sanger sequencing tracks of PCR products shown in (A) to the WT reference sequence. gRNAs used in the experiment are highlighted on the reference sequence with black boxes; their PAM sequences are shown with red boxes. All three RIG-I clones showed the same 58 bp deletion homozygously. MDA5 KO #1 and #3 had a homozygous 59 bp deletion; clone #5 had the same 59 bp deletion on one allele and a large insertion on the other allele. (C) Western blot analysis for RIG-I and MDA5 expression in HEK293 RIG-I, and MDA5 KO cells. Cells were transfected with 50 nM of gRNA to stimulate an IFNβ response and then harvested for protein extraction after 48 h. (D) Western blot analysis for MAVS expression in HEK293 WT and MAVS KO cells. Our KO strategy targeted the main isoform of MAVS (shown by arrow). Asterisk indicates nonspecific band. gRNA, guide RNA; HEK293, human embryonic kidney 293; IFNβ, interferon beta; KO, knockout; MAVS, mitochondrial antiviral signaling; MDA5, melanoma differentiation–associated gene 5; PAM, protospacer-adjacent motif; RIG-I, retinoic acid–inducible gene I; WT, wild-type. (TIF) [file pbio.2005840.s001.tif]

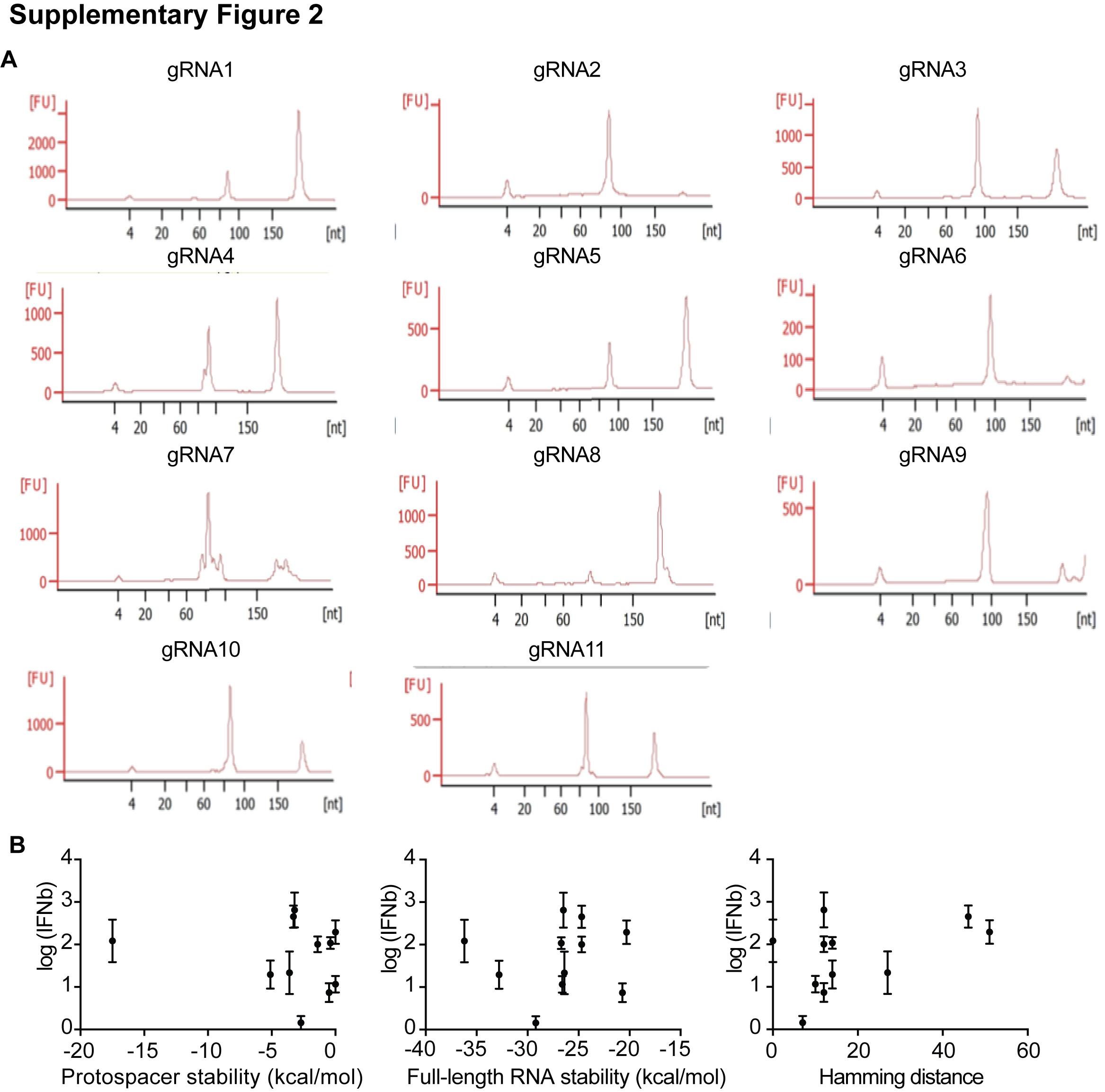

Supplement: S2 Fig — (A) Bioanalyzer results for gRNAs tested in Fig 3A. IVT gRNAs were denatured for 5 min at 70°C before analysis. (B) Correlation between IFNB1 activation and RNA stability or hamming distance, respectively. Predicted RNA secondary structure was calculated using Vienna RNA Fold [46]. Hamming distance reflects the extent to which the protospacer might interact with the gRNA constant region. The predicted secondary structure of the constant region in isolation was compared to the predicted secondary structure of the constant region when paired with the protospacer. The hamming distance between the dot-bracket notation–predicted secondary structure in each context is shown. gRNA, guide RNA; IFNβ, interferon beta; IFNB1, interferon beta 1; IVT, in vitro–transcribed. (TIF) [file pbio.2005840.s002.tif]

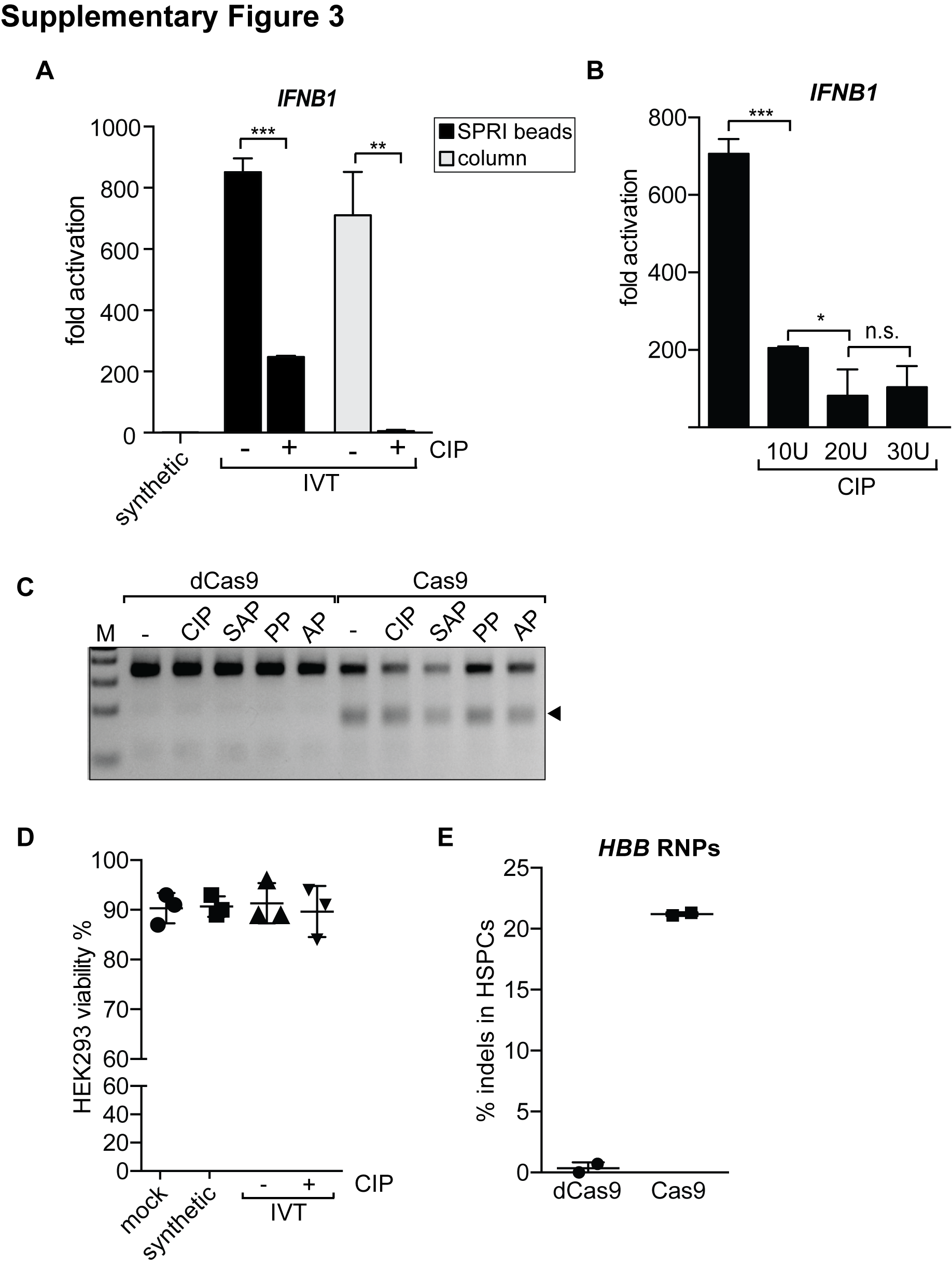

Supplement: S3 Fig — (A) qRT-PCR analysis of IFNB1 transcript levels in HEK293 cells transfected with synthetic, IVT, and CIP IVT gRNAs (gRNA1). After in vitro transcription and CIP-treatment, gRNAs were purified with SPRI beads or spin columns, respectively. Cells were harvested for RNA extraction 30 h after transfection with RNAiMAX transfection reagent. Average values of 3 biological replicates +/−SD are shown (B) qRT-PCR analysis of IFNB1 transcript levels in HEK293 cells transfected with IVT gRNA via RNAiMAX lipofection. IVT gRNAs were treated with 0, 10, 20, or 30 units (U) of CIP, respectively, before purification with SPRI beads. (C) T7E1 assay to determine cleavage efficiencies of phosphatase-treated IVT gRNA-RNPs targeting the BFP locus in HEK293T-BFP cells. HEK293T-BFP cells were nucleofected with Cas9/dCas9-RNPs and harvested after 24 h. PCR-amplified target DNA was heated, reannealed, and digested with T7E1 before gel electrophoresis. (D) Viability of HEK293 cells after transfection with gRNAs. Viability was determined using trypan blue exclusion test. (E) Editing outcome in primary HSPCs that were nucleofected with dCas9 or Cas9-IVT gRNA RNPs targeting the HBB locus. Amounts of indels were determined 24 h after transfection by PCR across the target site, followed by Sanger sequencing and TIDE analysis. Statistical significances were calculated by unpaired t test (*p < 0.05, ***p < 0.0001). The underlying data for this figure can be found in S1 Data. BFP, blue fluorescent protein; Cas9, CRISPR-associated 9; CIP, Calf intestine phosphatase; dCas9, nuclease-dead CRISPR-associated 9; gRNA, guide RNA; HEK293, human embryonic kidney 293; HBB, hemoglobin subunit beta; IFNB1, interferon beta 1; indel, insertion and deletion; IVT, in vitro–transcribed; n.s., not significant; qRT-PCR, quantitative real-time PCR; SPRI, solid-phase reversible immobilization; RNP, ribonucleoprotein; T7E1, T7 endonuclease 1. (TIF) [file pbio.2005840.s003.tif]
