# Supplementary material for: In vitro–transcribed guide RNAs trigger an innate immune response via the RIG-I pathway
Source: PLoS Biol. 2018 Jul 16;16(7):e2005840. doi: 10.1371/journal.pbio.2005840 (PMC6049001; doi:10.1371/journal.pbio.2005840)
Supplement: S1 Table — (DOCX) [file pbio.2005840.s004.docx]

**Supplementary Table 1: Primers for *in vitro* transcription**

| Name | F/R | Sequence 5’ to 3’ |
| --- | --- | --- |
| T7FwdAmp T7RevAmp | F | GGATCCTAATACGACTCACTATAG |
|  | R | AAAAAAGCACCGACTCGG |
| T7RevLong | R | AAAAAAGCACCGACTCGGTGCCACTTTTTCAAGTTGATAACGGACTAGCCTTATTTTAACTTGCTATTTCTAGCTCTAAAAC |
| T7FwdVar | F | GGATCCTAATACGACTCACTATA(G)-N_20_-GTTTTAGAGCTAGAA |
| HCV fwd | F | NCCTATAGTGAGTCGTATTA |
| HCV rev | R | AAAGGAAAGAAAAGGAAAAAAAGAGGAAAAAAAAAGGAGAAAAAAAAAAAAAAAAAAAAAAAAAAAAAAAAAAGAAAAAAAAAAAGGGAAAAAAACAGGATGGCCTATAGTGAGTCGTATTA |
