# Supplementary material for: In vitro–transcribed guide RNAs trigger an innate immune response via the RIG-I pathway
Source: PLoS Biol. 2018 Jul 16;16(7):e2005840. doi: 10.1371/journal.pbio.2005840 (PMC6049001; doi:10.1371/journal.pbio.2005840)
Supplement: S2 Table — gRNA, guide RNA. (DOCX) [file pbio.2005840.s005.docx]

**Supplementary Table 2: gRNA sequences**

| Name | Targeting | Sequence 5' to 3' |
| --- | --- | --- |
| RIG-I #1 | RIG-I Exon 2 | TCAGGCTGAGAAAAACAACA |
| RIG-I #2 |  | CACGGAACCAGCCTTCCTCC |
| MDA5 #1 | MDA5 Exon 1 | TGAGAAAGAAAGATGTCGAA |
| MDA5 #2 |  | CTGGATGTACATTTTCACCC |
| MAVS #1 | MAVS Exon 3 | TGTCTTCCAGGATCGACTGC |
| MAVS #2 |  | CCAGCCGGGCCGCCGCTGAA |
| gRNA1 | non/BFP | gctgaagcactgcacgccAT |
| gRNA2 | non | cttcagggtcagcttgccgt |
| gRNA3 | non | tgaagaagatggtgcgctcc |
| gRNA4 | non | cggtggtgcagatgaacttc |
| gRNA5 | non | cgcttccgcggcccgttcaa |
| gRNA6 | non | aatcgtatcgaaatctatcag |
| gRNA7 | non | ctggacgtagccttcgggca |
| gRNA8 | non | agaagtcgtgctgcttcatg |
| gRNA9 | non | ggagcgcaccatcttcttca |
| gRNA10 | non | cggcgccggcaacggcgccg |
| gRNA11 | non | acggaggctaagcgtcgcaa |
| HBB | HBB exon 1 | CTTGCCCCACAGGGCAGTAA |
| JAK2 | intronic | TCAGTTTCAGGATCACAGCT |
