# Supplementary material for: In vitro–transcribed guide RNAs trigger an innate immune response via the RIG-I pathway
Source: PLoS Biol. 2018 Jul 16;16(7):e2005840. doi: 10.1371/journal.pbio.2005840 (PMC6049001; doi:10.1371/journal.pbio.2005840)
Supplement: S3 Table — qRT-PCR, quantitative real-time PCR. (DOCX) [file pbio.2005840.s006.docx]

**Supplementary Table 3: qRT-PCR primers**

| Gene | F/R | Sequence 5’ to 3’ |
| --- | --- | --- |
| *GAPDH* | F | ATTCCACCCATGGCAAATTC |
|  | R | TGGGATTTCCATTGATGACAAG |
| *IFNB1* | F | ttcagtgtcagaagctcctgtgg |
|  | R | ctgcttaatctcctcagggatgtca |
| *ISG15* | F | ggcagcgaactcatcttt |
|  | R | cagcatcttcaccgtcag |
| *MDA5 (IFIH1)* | F | aggaggaactgttgacaattg |
|  | R | agtagctctcttacacctgattc |
| *RIG-I (DDX58)* | F | tggaccctacctacatcctg |
|  | R | tcagcctgaatatactgcac |
