# Supplementary material for: In vitro–transcribed guide RNAs trigger an innate immune response via the RIG-I pathway
Source: PLoS Biol. 2018 Jul 16;16(7):e2005840. doi: 10.1371/journal.pbio.2005840 (PMC6049001; doi:10.1371/journal.pbio.2005840)
Supplement: S4 Table — (DOCX) [file pbio.2005840.s007.docx]

**Supplementary Table 4: genomic PCR primers**

| Primer name | F/R | Sequence 5’ to 3’ |
| --- | --- | --- |
| RIG-I gPCR | F | GTGACAATAATCTCTCTGCAGATTC |
|  | R | TGGTACAATTCCTGTCCCTATTT |
| MDA5 gPCR | F | CTGTGGACAACCTCGTCATT |
|  | R | GTTCCTCCTCCATGCACTTATC |
| BFP gPCR | F | AGCTGGACGGCGACGTAAAC |
|  | R | ATGCGGTTCACCAGGGTGTC |
| HBB set 1 | F | CACTTAGACCTCACCCTGTG |
|  | R | TATGGGACGCTTGATGTTTTCT |
| HBB set 2 | F | TATGGGACGCTTGATGTTTTCT |
|  | R | CTCTGCCTATTGGTCTATTTTCCCA |
